# A community-based, cross-sectional study to assess interactions between income, nutritional status and enteric parasitism in two Brazilian cities: are we moving positively towards 2030?

**DOI:** 10.1186/s41043-021-00252-z

**Published:** 2021-06-07

**Authors:** Deiviane A. Calegar, Polyanna A. Bacelar, Kerla J. L. Monteiro, Jessica P. dos Santos, Andressa B. Gonçalves, Márcio N. Boia, Lauren H. Jaeger, Beatriz Coronato-Nunes, Filipe A. Carvalho-Costa

**Affiliations:** 1 Laboratório de Epidemiologia e Sistemática Molecular, Instituto Oswaldo Cruz, Fundação Oswaldo Cruz, Rio de Janeiro, Rio de Janeiro Brazil; 2 Escritório Técnico Regional - Fundação Oswaldo Cruz, Piauí, Rua Magalhães Filho, 519, Centro/Norte, Teresina, Piauí Brazil; 3 Laboratório de Biologia e Parasitologia de Mamíferos Silvestres Reservatórios, Instituto Oswaldo Cruz, Fundação Oswaldo Cruz, Rio de Janeiro, Rio de Janeiro Brazil; 4 Faculdade de Farmácia, Universidade Federal de Juiz de Fora, Rua José Lourenço Kelmer, s/n – Campus Universitário Bairro São Pedro, Juiz de Fora, Minas Gerais Brazil; 5 Faculdade de Medicina de Petrópolis (FMP)/ Centro Universitário Arthur Sá Earp Neto (UNIFASE), Rua Machado Fagundes, 326, Cascatinha, Petrópolis, Rio de Janeiro, Brazil

**Keywords:** Intestinal parasitism, Nutrition, Children

## Abstract

**Background:**

This study assessed the interactions between income, nutritional status and intestinal parasitism in children in Brazil.

**Methods:**

A cross-sectional study (n = 421 children aged 1 to 14 years living in the states of Piauí (rural communities in the city of Teresina) and Rio de Janeiro (rural and periurban communities in the city of Cachoeiras de Macacu) was performed in order to obtain income and anthropometric data, as well as fecal samples for parasitological analyses through the Ritchie technique.

**Results:**

Children infected with *Ascaris lumbricoides* had significantly lower means of height-for-age z scores (− 1.36 ± 0.75 vs. − 0.11 ± 1.02; *p* < 0.001), weight-for-age z scores (− 1.23 ± 0.74 vs. 0.09 ± 1.15; *p* = 0.001), and weight-for-height z scores (− 0.68 ± 0.44 vs. 0.23 ± 1.25; p = 0.006) when compared with uninfected children. Infection with hookworm was also associated with lower means of height-for-age z scores (− 1.08 ± 1.17 vs. − 0.12 ± 1.02; p = 0.015) and weight-for-age z scores (− 1.03 ± 1.13 vs. 0.08 ± 1.15; p = 0.012). Children infected with *Entamoeba coli* presented significantly lower means of height-for-age z scores (− 0.54 ± 1.02 vs. − 0.09 ± 1.02; p = 0.005) and weight-for-age z scores (− 0.44 ± 1.15 vs. 0.12 ± 1.15; p = 0.002). The multivariate multiple linear regression analysis showed that height-for-age z scores are independently influenced by monthly per capita family income (β = 0.145; p = 0.003), female gender (β = 0.117; p = 0.015), and infections with *A. lumbricoides* (β = − 0.141; p = 0.006) and *Entamoeba coli* (β = − 0.100; *p* = 0.043). Weight-for-age z scores are influenced by monthly per capita family income (β = 0.175; *p* < 0.001), female gender (β = 0.123; p = 0.010), and infections with *A. lumbricoides* (β = − 0.127; *p* = 0.012), and *Entamoeba coli* (β = − 0.101; *p* = 0.039). Monthly per capita family income (β = 0.102; *p* = 0.039) and female gender (β = 0.134; *p* = 0.007) positively influences mid upper arm circumpherence.

**Conclusions:**

Intestinal parasitism and low family income negatively influence the physical development of children in low-income communities in different Brazilian regions.

## Introduction

The first of the seventeen United Nations Sustainable Development Goals aims to reduce by at least half the proportion of people living in poverty by 2030. The second includes ending all forms of malnutrition, including meeting the internationally agreed targets for stunting and wasting in children under the age of five. The third goal includes ending epidemics of waterborne and neglected tropical diseases [1]. These three goals are interconnected and the dimensions they address—income, food and health—interact in a multi-causal feedback network.

In 1991, 67% of the Brazilian population lived in poverty (monthly per capita household income (MPCHI) less than half the Brazilian minimum wage) [2]. This proportion was reduced to 49% in 2000 and 34% in 2010. This year, large regional variation in the poverty rate was observed, with 56% in the Northeast, 53% in the North, 26% in the Central-West, 24% in the Southeast and 19% in the South [3]. More recent estimates show that the poverty rate fell from 26.5% in 2017 to 25.3% in 2018, still higher than in 2012 when the pre-recession rate was 22.8%. In addition, extreme poverty in Brazil last year reached its highest level since 2012, with 6.5% of the population—about 13.5 million people—with a monthly income below 40 USD [4].

The proportion of boys and girls aged 5–9 years with chronic malnutrition characterized by stunting (height deficit) was reduced respectively from 29.3% and 26.7% in 1975 to 7.2% and 6.3% in 2009 [5]. In parallel, the frequency of weight deficit in boys and girls aged 5–9 years dropped respectively from 5.7% and 5.4% to 4.3% and 3.9% in the same period [5]. The latest national-based survey also showed important regional differences, with higher prevalence rates of malnutrition in northern Brazil, lower rates in the South and similar rates close to the national average in the Northeast, Southeast and Central-West [5]. The specific mortality rate due to diarrheal disease in children under five years in Brazil was reduced from 22/100,000 children in 2000 to 5.5/100,000 in 2013. Regarding the regions, these rates in 2013 were 12.5 in the North, 8.3 in the Northeast, 6 in the Central-West, 2.3 in the Southeast and 2.1 in the South [6].

Soil-transmitted helminths (STHs), including *Ascaris lumbricoides*, hookworm (*Necator americanus* and *Ancylostoma duodenale*) and *Trichuris trichiura*, are notable for the potential severity of infection, which can cause intestinal obstruction (ascariasis), severe anemia (hookworm disease), and rectal prolapse and dysentery (trichuriasis) [1, 7]. Deficits in the physical and cognitive development of children are insidious and have chronic effects [8].

Despite the scarcity of country-based data on the burden of different soil-transmitted helminthiases (STHs) in Brazil, an analysis with mathematical modeling of secondary data estimates that the prevalence rates of ascariasis, hookworm infection and trichuriasis are 3.6%, 1.7% and 1.4% respectively, after scaling up of preventive chemoprophylaxis (mass drug administration (MDA)) and primary health care in the country [9]. However, in some rural and urban communities with poor sanitation and practicing open defecation, prevalence rates can be significantly higher [10–13]. Pathogenic species of protozoa inhabit the human digestive tract, including *Giardia duodenalis* and *Entamoeba histolytica*. These organisms are also associated with chronic nutrient spoliation and affect the physical and cognitive development of children [11, 14, 15]. *G. duodenalis* causes about 280 million symptomatic infections per year worldwide [16], and in low- and middle-income countries, the prevalence of giardiasis can reach up to 30% [17]. The transmission dynamics of giardiasis is complex, and it can be considered a zoonotic disease [17, 18]. It is estimated that amebiasis is associated with more than 55,000 deaths per year and that morbidity due to this parasite leads to the loss of 2.2 million disability-adjusted life years (DALYs) [19].

There are no policies to control intestinal protozoan infections, and anti-STH MDA campaigns have made protozoa even more neglected [7, 8, 15]. In developing countries, this has influenced the etiologic profile of parasitic enteric infections towards a higher frequency of protozoa detection in coproparasitological surveys in recent decades [20–22]. The human intestine also hosts presumably non-pathogenic protozoans, such as *Entamoeba coli*, *Endolimax nana*, *Iodamoeba butschlii*, *Blastocystis hominis* and *Dientamoeba fragilis* [23]. Prevalence rates of infections with organisms such as *Entamoeba coli* have been considered indicators of environmental contamination with fecal matter in poor sanitation scenarios [21].

Regarding the impact of intestinal parasitism on the nutritional status of children, the literature presents conflicting results. This assessment is difficult due to the failure to consider an important confounding factor, family income, which may be very heterogeneous in some populations. According to the National Household Budget Survey [5], family income strongly influences the anthropometric parameters used to assess children's nutritional status in Brazil.

## Objective

The study aim was to evaluate the interactions between income, nutritional status and intestinal parasitism in children living in periurban communities in two states in the Northeast and Southeast regions of Brazil.

## Materials and methods

### Description of the study areas and population

**Fig. 1:**
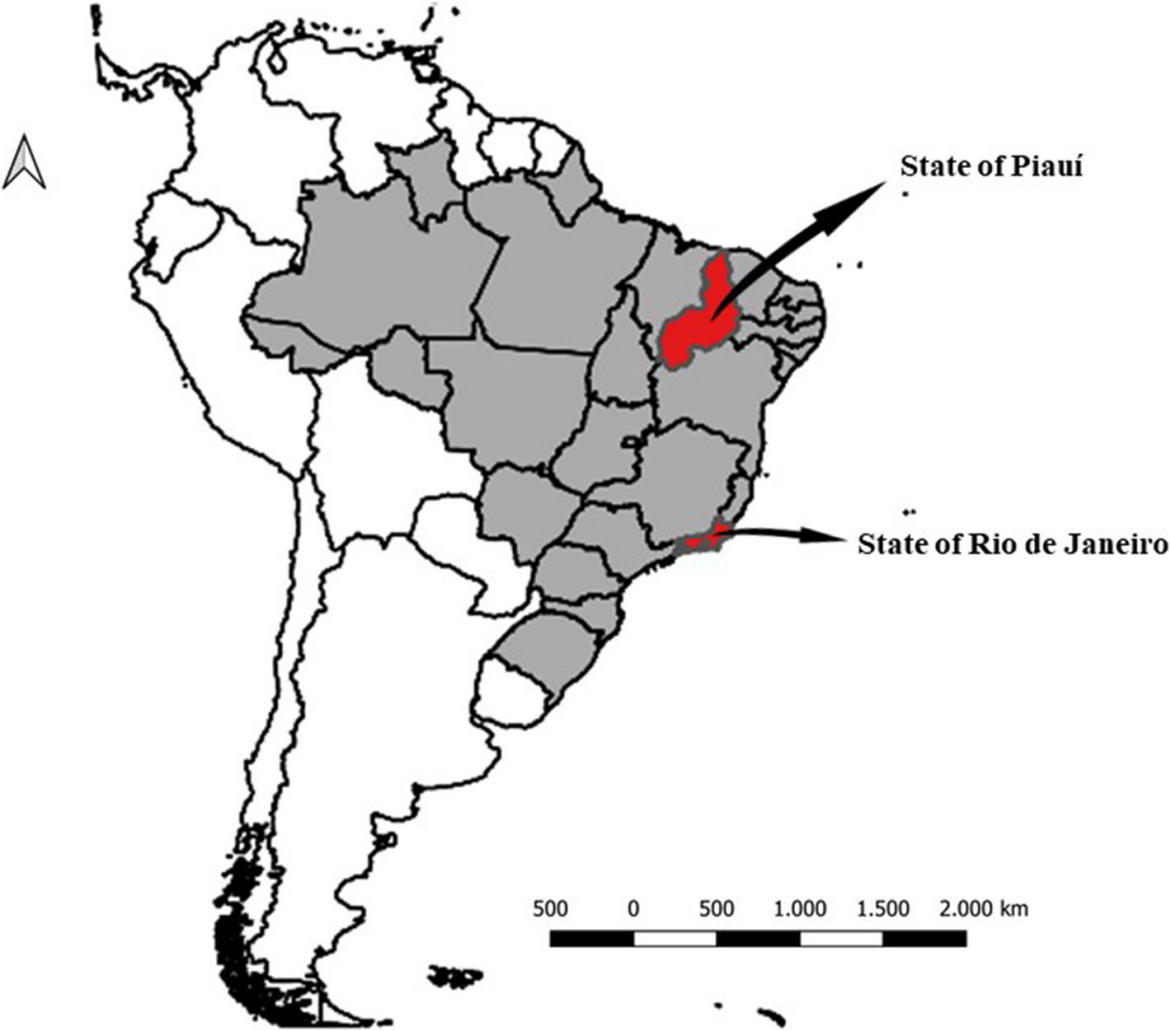
Geographic localization of the studied municipalities: Teresina, in the state of Piauí and Cachoeiras de Macacu, in the state of Rio de Janeiro

The study was carried out in August–September 2017 in the city of Teresina (TER) in the state of Piauí (Northeast macroregion) and from May 2017 to May 2019 in Cachoeiras de Macacu (CAM), in the state of Rio de Janeiro (Southeast macroregion) (see map in Fig. 1). In TER, two periurban communities with rural characteristics involved in the agrarian reform process (Camp 8 de Março and Settlement 17 de Maio) were studied, whose livelihood is obtained through family farming. In CAM, two urbanized districts (Papucaia and Ribeira) were studied, in addition to a community with rural characteristics (Marubaí). The communities studied in TER have a transitional tropical climate within the ecotonal zone called Mata de Cocais, which is situated in a transition zone between the Amazonian biomes (in the West), the Cerrado (in the South) and the Caatinga (in the East). In these communities, a high proportion of the population practice open defecation. In CAM, the areas studied have a tropical climate and are located in remnant areas of the Atlantic Forest, but with a high degree of deforestation for agriculture and livestock. The state of Rio de Janeiro has a higher Human Development Index (0.794) when compared to Piauí (0.690) [3].

### Study design and statistical analyses

**Fig. 2:**
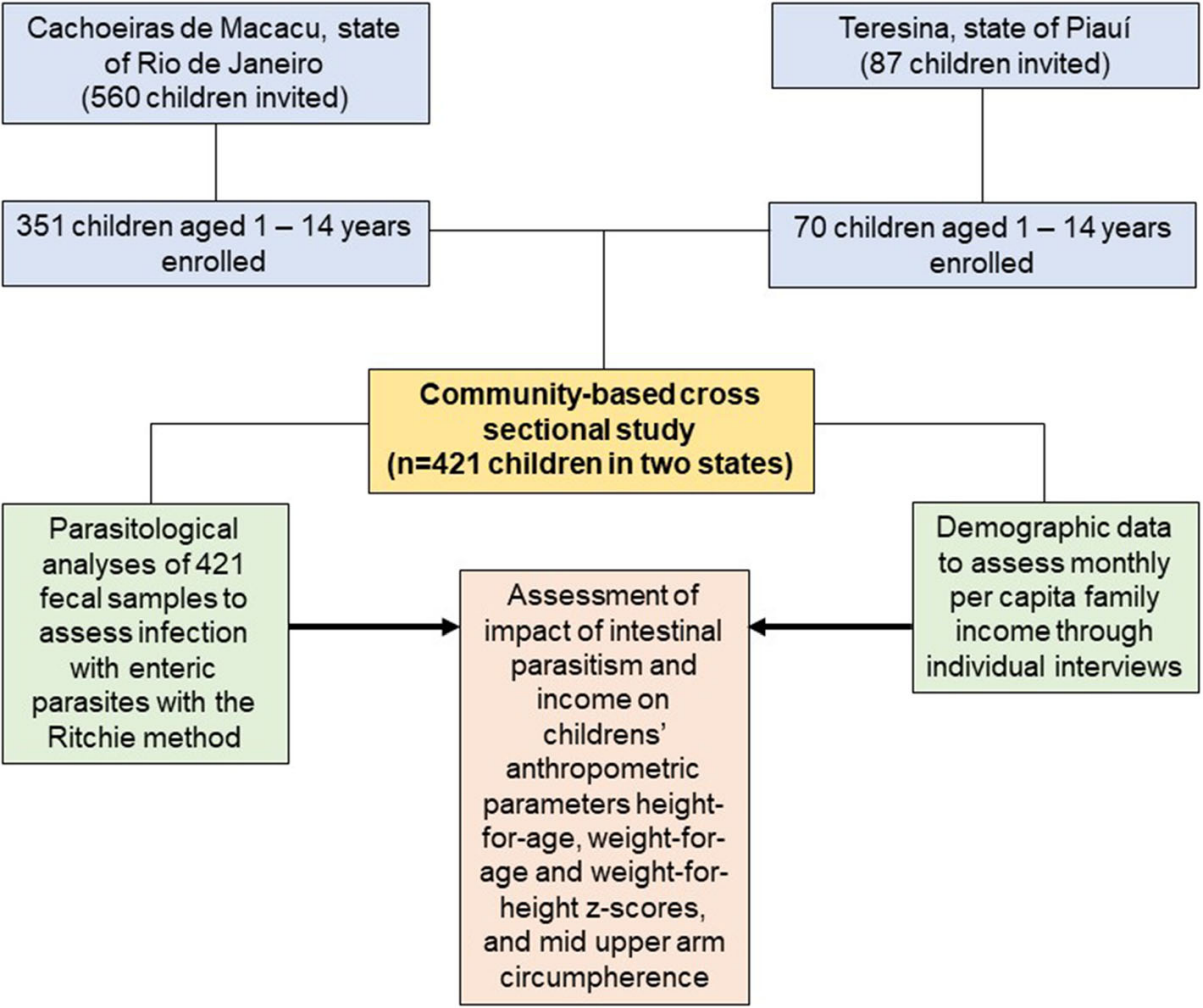
Descriptive flowchart of the general lines of the study

A community-based cross-sectional study was performed to obtain sociodemographic, anthropometric and parasitological data from 421 children living in TER (*n* = 70) and CAM (*n* = 351) (Fig. 2). All children aged 1 to 14 years old who lived in the studied communities were invited to participate in the study, and those who had chronic diseases were excluded due to the influence of baseline conditions on nutritional status. Sample sizes were not calculated, as we tried to include all children in the communities, to whom the pots for fecal collection were distributed. The rates of adherence and return of the fecal sample were approximately 50%, not significantly different between communities.

Body weight was recorded with a portable electronic scale, to the nearest 100 g. Children wore minimal clothing and were barefoot. Height was measured using an anthropometer to the nearest 0.1 cm. Z scores (standard deviation scores) of height for age (HAZ), weight for age (WAZ) and weight for height (WHZ) were assessed using the NutStat Module on EpiInfo 2000 version 3.2.2 using the CDC growth charts [24]. Stunting, underweight and wasting were defined by values equal or below − 2 for HAZ, WAZ and WHZ respectively. The mid upper arm circumference (MUAC) of the right arm was assessed with a flexible measuring tape, in the midpoint between the acromion and the olecranon processes. This study followed the STROBE guidelines for cross-sectional studies.

After checked for normality, z score means of anthropometric parameters in children infected and uninfected with different parasites, living in distinct states and belonging to distinct genders were compared with Student's t tests. The correlation between the monthly per capita family income (MPCFI) of the families to which the children belonged and the anthropometric parameters was evaluated by simple linear regression. The rate of positivity for distinct parasites in distinct income groups was compared through the Fisher's exact test. Independent variables that significantly influenced anthropometric parameters were selected and multivariate analysis by multiple linear regression was performed in order to assess the influence of enteric parasitic infections on nutritional status, considering family income as the main confounding factor. In multiple linear regression, the z scores of the anthropometric indicators were considered dependent variables, and the variables selected in the bivariate analyses were tested to evaluate interactions, including gender and state. In all analyses, a *p* value of < 0.05 was used to establish statistical significance.

### Laboratory procedures

Fecal samples were analyzed using the Ritchie technique [25] for the detection of helminth eggs and protozoan cysts. Briefly, 10% fecal suspensions were percolated in gauze and centrifuged (2500 rpm for 2 min). The supernatant was discarded, and 7 mL of distilled water, 3 mL of ethyl acetate and 1 drop of detergent were added. After further centrifugation and discarding of the supernatant, the pellet was analyzed by light microscopy [25].

## Results

Table 1 presents the characteristics of the 421 children included in the study. The rates of chronic malnutrition (HAZ < − 2) in RJ and PI were 3.1% and 5.7%, respectively, the rates of low weight (WAZ < − 2) were 3.7% and 4.3%, and the rates of wasting (WHZ < − 2) were 11% and 0%, respectively. The prevalence rate of obesity (WAZ > 2) was 6.6% in RJ and 2.9% in PI. The proportion of children living in families with MPCHI < 45 USD (poverty) was 39.3% in RJ and 67% in PI.

**Table 1:** Demographic characteristics and nutritional status of children participating in the study in the states of Piauí and Rio de Janeiro, Brazil, 2017 and 2018

	Piauí n (%)	Rio de Janeiro n (%)
**Gender**		
Male	39 (55.7)	190 (54.1)
Female	31 (44.3)	161 (45.9)
**Age group**		
0–5	21 (30)	123 (35)
6–10	27 (38.6)	156 (44.4)
11–14	22 (31.4)	72 (20.5)
Per capita monthly family income (USD*)		
0–45	47 (67.1)	138 (39.3)
> 45	23 (32.9)	213 (60.7)
**Nutritional status**		
**Heigth-for-age z score**		
< − 2	4 (5.7)	11 (3.1)
− 2 to + 2	63 (90)	330 (94)
> + 2	3 (4.3)	10 (2.8)
**Weigth-for-age z score**		
< − 2	3 (4.3)	13 (3.7)
− 2 to + 2	65 (92.9)	315 (89.7)
> + 2	2 (2.9)	23 (6.6)
**Weigth-for-height z score**		
< − 2	-	11 (6.5)
− 2 to + 2	45 (95.7)	141 (83.9)
> + 2	2 (4.3)	16 (9.5)

* USD 1 = BRL 4

**Fig. 3:**
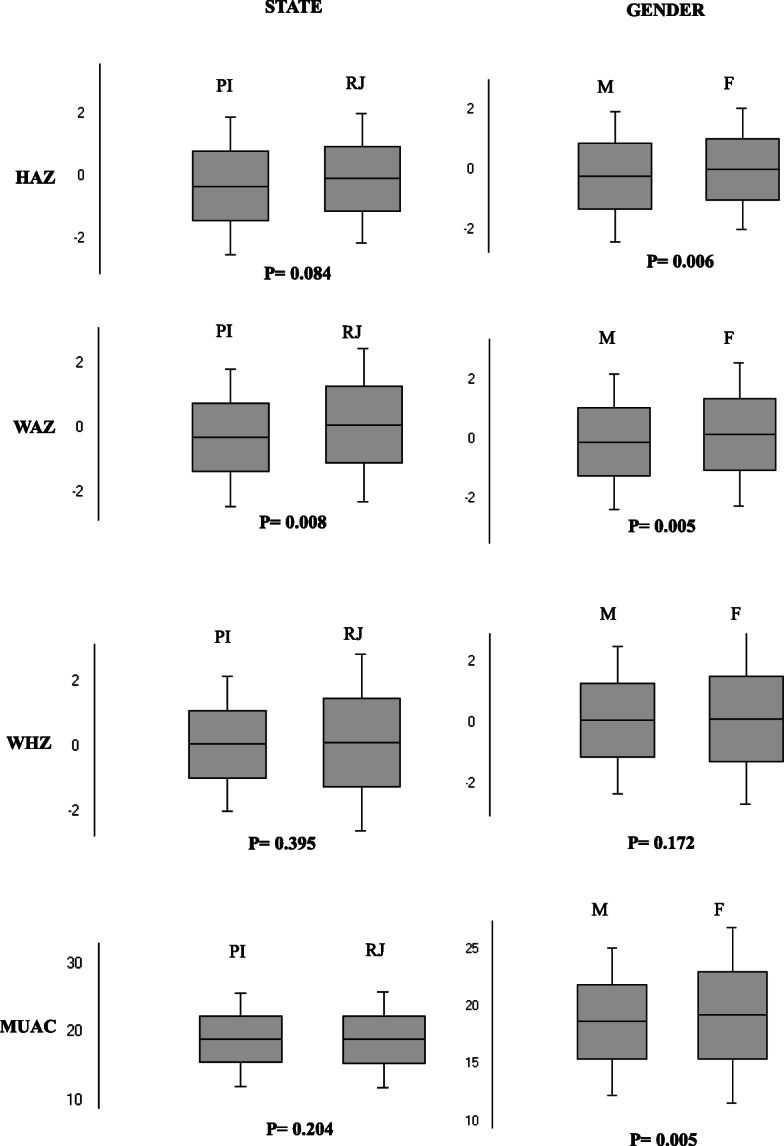
Anthropometric parameters, z scores, of studied children by state and gender, Piauí and Rio de Janeiro, Brazil, 2017 and 2018

Figure 3 shows the comparison of z score means of anthropometric parameters by state and gender. The WAZ mean was lower among children living in PI than in RJ (− 0.27 ± 1.06 vs. 0.13 ± 1.17; p = 0.008). The HAZ and WAZ means were lower among boys than among girls (− 0.26 ± 1.07 vs. 0.01 ± 0.96; p = 0.006, and − 0.08 ± 1.22 vs. 0.24 ± 1.18; p = 0.005, respectively).

**Fig. 4:**
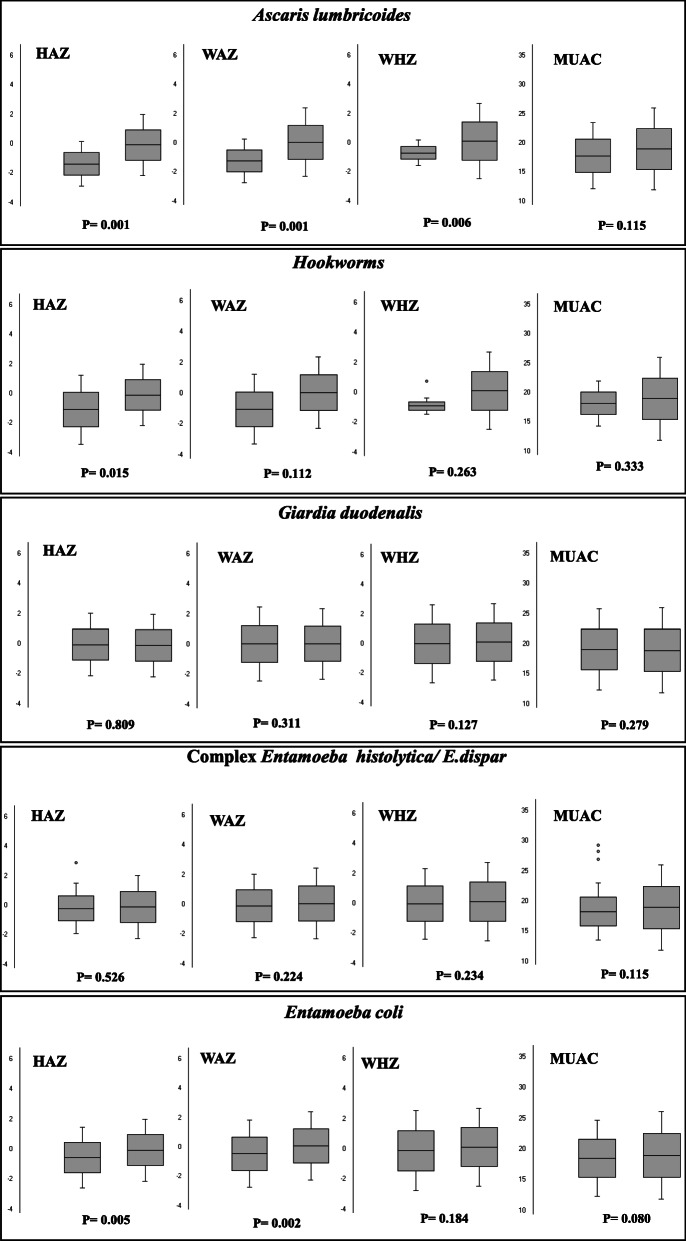
Anthropometric parameters, z scores, according to intestinal parasite infection status in children of Teresina and Cachoeiras de Macacu, 2017 and 2018

The comparison of means in children infected and uninfected by different parasites is represented in Fig. 4. Children infected with *A. lumbricoides* had significantly lower means of HAZ (− 1.36 ± 0.7 vs. − 0.11 ± 1.02; *p* < 0.001), WAZ (− 1.24 ± 0.75 vs. 0.09 ± 1.15; *p* = 0.001), and WHZ (− 0.68 ± 0.45 vs. 0.23 ± 1.26; *p* = 0.006) when compared to uninfected children. Infection with hookworm was also associated with lower means of HAZ (− 1.08 ± 1.17 vs. − 0.12 ± 1.02; *p* = 0.015) and WAZ (− 1.03 ± 1.13 vs. 0.08 ± 1.15; *p* = 0.012). Children infected with *Entamoeba coli* presented significantly lower means of HAZ (− 0.54 ± 1.01 vs. − 0.09 ± 1.02; *p* = 0.005) and WAZ (− 0.44 ± 1.15 vs. 0.12 ± 1.15; *p* = 0.002). No significant differences in nutritional status were found among children infected with *E. histolytica* / *E. dispar* or *G. duodenalis*.

Table 2 presents the prevalence rates of infection by different intestinal parasites and other characteristics. Significantly higher positivity rates were observed in the state of Piauí when compared with Rio de Janeiro for *A. lumbricoides* (5.7% vs. 1.4%; p = 0.046), hookworm (8.6% vs. 0.3%; *p* < 0.001), *Entamoeba coli* (28.6% vs. 7.4%; *p* < 0.001) and *Hymenolepis nana* (2.9% vs. 0%; p = 0.027). Children belonging to families living in poverty (MPCHI < 45 USD) presented higher positivity rates for *A. lumbricoides* (3.8% vs. 0.8%; *p* = 0.047), *Entamoeba coli* (17.3% vs. 5.9%, *p* < 0.001) and *G. duodenalis* (15.1% vs. 6.4%, *p* = 0.004).

**Table 2:** Frequency and distribution of infection with distinct intestinal parasites by gender, age group, income group and state, in children in the states of Piauí and Rio de Janeiro, Brazil, 2017 and 2018

Characteristic	***Ascaris lumbricoides***	Hookworms	***E. histolytica / E. dispar***	***Giardia duodenalis***	***Entamoeba coli***
**Gender**					
Male (*n* = 229)	8 (3.5%)	7 (3.1%)	38 (16.6%)	23 (10%)	28 (12.2)
Female (*n* = 192)	1 (0.5%)	-	27 (14.1%)	20 (10.4%)	18 (9.4)
*p* value	0.044	0.017	0.501	1.000	0.433
**Age group (years)**					
0–5 (*n* = 144)	3 (2.1%)	-	20 (13.9%)	14 (9.7%)	14 (9.7%)
6–10 (*n* = 183)	3 (1.6%)	5 (2.7%)	34 (18.6%)	20 (10.9%)	23 (12.6%)
11–14 (*n* = 94)	3 (3.2%)	2 (2.1%)	11 (11.7%)	9 (9.6%)	9 (9.6%)
*p value*	0.796	0.232	0.746	0.932	0.993
Income (MPCHI*, USD**)					
≤ 45 (*n* = 185)	7 (3.8%)	4 (2.2%)	35 (18.9%)	28 (15.1%)	32 (17.3%)
> 45 (*n* = 236)	2 (0.8%)	3 (1.3%)	30 (12.2%)	15 (6.4%)	14 (5.9%)
*p value*	0.047	0.704	0.102	0.004	< 0.001
**State**					
Piauí (*n* = 70)	4 (5.7%)	6 (8.6%)	7 (10%)	8 (11.4%)	20 (28.6%)
Rio de Janeiro (*n* = 351)	5 (1.4%)	1 (0.3%)	58 (16.5%)	35 (10%)	26 (7.4%)
*p* value	0.046	< 0.001	0.206	0.669	< 0.001
**Total (*n*** **= 421)**	9 (2.1%)	7 (1.7%)	65 (15.4%)	43 (10.2%)	46 (10.9%)

* *MPCHI* monthly per capita house income; ** USD 1 = BRL 4

As shown in Table 3, the multivariate multiple linear regression analysis model showed that HAZ is independently influenced by MPCHI (β = 0.145; *p* = 0.003), female gender (β = 0.117; p = 0.015), and infections with *A. lumbricoides* (β = − 0.141; *p* = 0.006) and *Entamoeba coli* (β = − 0.100; p = 0.043). Similarly, WAZ is influenced by MPCHI (β = 0.175; *p* < 0.001), female gender (β = 0.123; *p* = 0.010), and infections with *A. lumbricoides* (β = − 0.127; *p* = 0.012) and *Entamoeba coli* (β = − 0.101; *p* = 0.039). MPCHI (β = 0.102; *p* = 0.039) and female gender (β = 0.134; p = 0.007) positively influences MUAC.

**Table 3:** Multiple linear regression analysis of anthropometric parameters, z scores, by infections with *Ascaris lumbricoides*, hookworms and *Entamoeba coli*; monthly per capita house income, gender and state of children in Piauí, Rio de Janeiro, Brazil, 2017 and 2018

Independent variables	HAZ		WAZ		WHZ		MUAC	
	Coefficient	*p* value	Coefficient	*p* value	Coefficient	*p* value	Coefficient	*p* value
*Ascaris lumbricoides*	− 0.141	0.006	− 0.127	0.012	− 0.089	0.243	− 0.047	0.363
Hookworms	− 0.031	0.552	− 0.027	0.599	− 0.023	0.772	0.004	0.940
*Entamoeba coli*	− 0.100	0.043	− 0.101	0.039	− 0.064	0.372	− 0.059	0.245
MPCHI *	0.145	0.003	0.175	< 0.001	0.100	0.150	0.102	0.039
Gender	0.117	0.015	0.123	0.010	0.070	0.314	0.134	0.007
State	0.011	0.825	0.054	0.283	0.005	0.948	0.025	0.635

* *MPCHI* monthly per capita house income

## Discussion

In this study, we assessed some socioenvironmental variables affecting the nutritional status of children living in two periurban areas in Brazil, focusing on income and enteric infections. The main findings were the influence of MPCHI and infection with some species of intestinal parasites on the nutritional parameters evaluated. These findings reveal the vulnerability of children living in poverty in periurban communities in the states of Rio de Janeiro and Piauí.

Regarding income, many of the children studied live in families whose MPCHI is less than 40 USD per month, which defines extreme poverty in Brazil. The data suggest that raising family income through minimum income programs positively affects children's weight, height and arm circumference, by improving access to food. Higher family income positively influenced both the chronic malnutrition indicator HAZ as well as the nonspecific parameter WAZ, besides MUAC, demonstrating that the effects of economic poverty on nutritional status are felt in both short and long term.

Brazil substantially reduced the proportion of people living in poverty in recent years. The results of this study suggest the need to maintain income policies as a tool to fight poverty in a time of economic recession that challenges most social programs implemented in recent decades. In this context, the current recessive cycle of the Brazilian economy, associated with rising unemployment, austerity, and a reduction in family income, may currently be undermining the advances afforded by Brazil in reducing the rates of child malnutrition achieved in recent decades, and constituting an unfavorable framework for meeting 2030 agenda goals.

Despite the low prevalence of ascariasis presented by the communities, it was demonstrated that this infection significantly correlates with worse nutritional status of the children included in the research. Despite infection with *A. lumbricoides* being focally present in a few children—confirming the current trend of reducing the prevalence of STHs in Brazil—it affects negatively the anthropometric parameters studied.

Some studies have evaluated the influence of STHs on children's nutritional status in developing countries. In Sri Lanka, despite no relationship being found between the presence of ascariasis and undernutrition, infections with high parasite load were associated with decreased values of WHZ [26]. In Vietnam, ascariasis influenced negatively the serum concentration of vitamin A in an infection intensity dependent way [27]. In Kenya, STH in preschool children was associated with vitamin A and iron deficiency [28]. In northwestern Ethiopia, no association was found between intestinal helminthic infections and nutritional status [29]. Among Venezuelan Amerindians, enteric helminthic infections were significantly associated with lower HAZ and WHZ [30]. In Mexico, an association between ascariasis and malnutrition in economically poor communities was demonstrated [31]. In Cameroon, children infected with STHs had significantly lower HAZ averages, with infection by more than one species being even more deleterious [32]. Hookworm and *A. lumbricoides* were associated with lower values of HAZ, WAZ and WHZ in Nigeria [33, 34]. In Chad, there was an association between *Hymenolepis nana* infection and malnutrition [35]. In Brazil, stunting was associated with ascariasis infection among children and adolescents [36]. On the other hand, two studies in Brazil demonstrated that the only enteric parasite associated with lower values of anthropometric parameters was *G. duodenalis* [15, 37]. Taken together, these data demonstrate how intestinal parasitism is a factor guiding the possibilities for the full physical development of children in developing countries.

This study demonstrated that infection with *Entamoeba coli* also influences the evaluated anthropometric parameters. *Entamoeba coli* is considered a non-pathogenic protozoan that commensally inhabits the human intestinal tract. Nevertheless, some studies have explored the potential of *Entamoeba coli* to affect bowel function. Recently, it was demonstrated that Mexican children infected with *Entamoeba coli* or *A. lumbricoides* were more likely to have higher levels of stool leucocytes than uninfected children [38], pointing to the possibility of intestinal inflammatory activity triggered by these parasites. *Entamoeba coli* can also be considered a marker of inadequate sanitary conditions, denoting greater exposure to fecal pathogens. Thus, it could be interpreted that, in this study, *Entamoeba coli*-positive children would have a higher frequency of other intestinal infections that would influence their nutritional status. In Bolivia, it has been shown that children living in a poorer scenario have a higher prevalence rate of *Entamoeba coli* infection and have worse nutritional status [39].

Another finding of the study was the influence of gender on nutritional status, showing that males presented lower values of the nutritional parameters evaluated. The National Household Budget Survey conducted in 2008–2009 showed similar results, with higher frequencies of weight and height deficits among boys [5].

Data show the interaction between income, nutritional status and enteric parasitic infections in children living in periurban areas in two Brazilian capitals, one in the Northeast and one in the Southeast. The results point to the need to improve both the income of families living in poverty and the sanitation scenario in these communities, 10 years before the year 2030, which is the horizon for the achievement of Sustainable Development Goals set by the United Nations.

## Data Availability

Data available on request due to privacy/ethical restrictions. The data that support the findings of this study are available on request from the corresponding author, Carvalho-Costa FA. The data are not publicly available because they contain information that could compromise the privacy of research participants.

## Abbreviations

MPCHI: Monthly per capita household income
STHs: Soil-transmitted helminthiases
MDA: Mass drug administration
TER: Teresina
CAM: Cachoeiras de Macacu
HAZ: Height for age
WAZ: Weight for age
WHZ: Weight for height
MUAC: Mid upper arm circumference
MPCFI: Monthly per capita family income
